# What effect have commissioners’ policies for body mass index had on hip replacement surgery?: an interrupted time series analysis from the National Joint Registry for England

**DOI:** 10.1186/s12916-023-02899-3

**Published:** 2023-06-13

**Authors:** Joanna McLaughlin, Ruth Kipping, Amanda Owen-Smith, Hugh McLeod, Samuel Hawley, J. Mark Wilkinson, Andrew Judge

**Affiliations:** 1 Musculoskeletal Research Unit, Translational Health Sciences, Bristol Medical School, University of Bristol, Learning and Research Building, Level 1, Southmead Hospital, Bristol, BS10 5NB UK; 2 Population Health Sciences, Bristol Medical School, University of Bristol, Bristol, BS8 2PS UK; 3 National Institute for Health and Care Research Applied Research Collaboration West (NIHR ARC West), University Hospitals Bristol NHS Foundation Trust, Bristol, BS1 2NT UK; 4 Department of Oncology and Metabolism, The Mellanby Centre for Musculoskeletal Research, University of Sheffield, Metabolic Bone Unit, Sorby Wing, Northern General Hospital, Sheffield, UK; 5 National Institute for Health and Care Research Bristol Biomedical Research Centre, University Hospitals Bristol and Weston NHS Foundation Trust and University of Bristol, Bristol, UK; 6 Nuffield Department of Orthopaedics, Rheumatology and Musculoskeletal Sciences (NDORMS), University of Oxford, Oxford, UK

**Keywords:** Hip replacement, Obesity, Epidemiology, Osteoarthritis, Commissioning, Health policy

## Abstract

**Background:**

Despite their widespread use, the impact of commissioners’ policies for body mass index (BMI) for access to elective surgery is not clear. Policy use varies by locality, and there are concerns that these policies may worsen health inequalities. The aim of this study was to assess the impact of policies for BMI on access to hip replacement surgery in England.

**Methods:**

A natural experimental study using interrupted time series and difference-in-differences analysis. We used National Joint Registry data for 480,364 patients who had primary hip replacement surgery in England between January 2009 and December 2019. Clinical commissioning group policies introduced before June 2018 to alter access to hip replacement for patients with overweight or obesity were considered the intervention. The main outcome measures were rate of surgery and patient demographics (BMI, index of multiple deprivation, independently funded surgery) over time.

**Results:**

Commissioning localities which introduced a policy had higher surgery rates at baseline than those which did not. Rates of surgery fell after policy introduction, whereas rates rose in localities with no policy. ‘Strict’ policies mandating a BMI threshold for access to surgery were associated with the sharpest fall in rates (trend change of − 1.39 operations per 100,000 population aged 40 + per quarter-year, 95% confidence interval − 1.81 to − 0.97, *P* < 0.001). Localities with BMI policies have higher proportions of independently funded surgery and more affluent patients receiving surgery, indicating increasing health inequalities. Policies enforcing extra waiting time before surgery were associated with worsening mean pre-operative symptom scores and rising obesity.

**Conclusions:**

Commissioners and policymakers should be aware of the counterproductive effects of BMI policies on patient outcomes and inequalities. We recommend that BMI policies involving extra waiting time or mandatory BMI thresholds are no longer used to reduce access to hip replacement surgery.

**Supplementary Information:**

The online version contains supplementary material available at 10.1186/s12916-023-02899-3.

## Background

Hip replacement is a common surgical procedure that is highly effective at reducing pain and improving functional outcomes in patients with end-stage hip osteoarthritis where non-surgical measures have failed to provide adequate improvement [1]. In countries of the Organization for Economic Cooperation and Development (OECD), the hip replacement rate increased by 22% from 2009 to reach a rate of 174 per 100,000 in 2019 [2]. One in 10 people in the UK can expect to receive a hip replacement at some point in their lifetime [3], and over 100,000 procedures were performed in 2019 in England and Wales [4]. Demand is increasing with an ageing population and rising levels of obesity [5]; even before the delays in access to surgery arising from the COVID-19 pandemic, more than half a million people were on the waiting list for elective trauma and orthopaedics in England and Wales [6].

Pathways to surgery across the National Health Service (NHS) are increasingly incorporating ‘health optimisation’ interventions for patients to improve their health before surgery, and these may include weight loss. There is variation in the approach chosen by commissioning localities; their policies range from recommendations that patients are given advice to lose weight to the use of extra waiting time or mandatory body mass index (BMI) thresholds for referral to surgery [7, 8]. Employing the ‘teachable moment’ of surgery to engage a patient with weight loss is intended to reduce a patient’s need for surgery, improve surgical outcomes and trigger lasting lifestyle changes [9, 10]. Where BMI is used to limit access to surgery, health optimisation presents an interplay between rationing for resource preservation and health improvement [11–13]. Despite guidance that surgical commissioning policies should not be based on factors such as a patient’s weight [14], by 2021, around 70% of England’s NHS clinical commissioning groups (CCGs) restricted access to joint replacement based on BMI [15].

Evaluations of some holistic approaches to supporting patients with health improvement in the pre-operative period have shown promising results [16–18], but the impact of BMI threshold use to limit access to surgery has not been well-examined. We have recently published analyses of knee replacement surgery rates in England that indicate BMI policies are associated with drops in the rate of surgery and with widening inequalities in patients [19]. Our aim in this study was to understand the impact of different severities of BMI policy on inequalities and patient access to elective hip replacement surgery in England. Using data from the National Joint Registry, we used a natural experimental study design with interrupted time series analyses to model the impact introduction of these policies has had on trends in rates of elective hip replacement surgery. We examined the difference in outcomes between CCGs with and without BMI policies. Our a priori hypothesis [20] was that stricter policy introduction would be associated with a greater reduction in the rate of surgery.

## Methods

### Study design

We used a quasi-experimental natural experiment study design [21–23]. We evaluated the impact of the introduction of CCG health optimisation policies on trends before and after the implementation of the intervention. The timing of the introduction of health optimisation policies varied by CCG. Whilst CCGs ceased as organisations in July 2022 and were replaced by Integrated Care Boards [24], this paper uses data relating to commissioning by CCGs prior to this change.

### Data source

We used data from the National Joint Registry for England, Wales, Northern Ireland and the Isle of Man (NJR). The NJR contains data on all publicly and privately funded hip replacement operations and includes 2 million patients since 2003, covering 96% of primary hip replacements [4]. It is mandatory for surgeons and their hospitals to register all hip replacement activity in the NJR, whether the procedures are funded by the NHS or independently. The NJR contains anonymised patient data on age, gender and date of procedure. Information on the patient’s residential area, as defined by the 2011 census Lower Layer Super Output Areas (LSOA), is also available. LSOAs are defined as geographical areas of similar population sizes, with an average of 1500 residents [25]. We used the dataset prepared for the NJR’s 2019 annual report [26] which therefore did not require further cleaning or coding. We used data provided by the Office for National Statistics (ONS) to identify the LSOAs nested in each CCG locality [27]. As a measure of socioeconomic deprivation, we used the index of multiple deprivation (IMD) score, a relative measure of deprivation based on LSOAs. We used the IMD rank for a patient’s LSOA and categorised patients into quintiles based on the national ranking of local areas, with quintile 1 being the most deprived group and quintile 5 being the least deprived group. Patient-reported outcome measures (PROMS) comprising pre- and post-operative Oxford Hip Score questionnaire data were linked to the NJR dataset at the patient level. The Oxford Hip Score is a validated hip-specific measure scored 0–48 with 0 indicating the most severe symptoms [28]. Information on relevant CCG policy content, introduction and cessation dates was gathered in July 2021 through the collection of policy documentation from CCG websites supplemented with Freedom of Information requests to each CCG [8].

### Participants and inclusion criteria

The study sample consisted of 849,686 patients who had a primary hip replacement in England between January 2009 and December 2019 recorded in the NJR. The inclusion criteria were patients aged 40 + years with osteoarthritis as a primary reason for surgery.

### Outcome measure

The primary outcome was the rate of provision of primary hip replacement for each CCG. For each annual quarter in each CCG, rates (expressed as per 100,000 persons aged 40 +) of surgery were determined by aggregating the number of eligible primary hip replacement procedures in the CCG locality (numerator) and using the aggregated ONS count of the population aged 40 + years living in each of these CCG localities in 2019 as the denominator [29].

The secondary outcome measures were the proportion of independently funded operations, the proportion of operations performed in patients with obesity (BMI 30 +) and the mean pre-operative Oxford Hip Score. For BMI and Oxford Hip Score calculations, only the individual records with a BMI record in the range of 12 to 60 kg/m^2^ or a recorded Oxford Hip Score were retained respectively. Further detail on BMI and PROMS data reported to the registry is given in the NJR annual report [26].

### Intervention

The intervention was the date the CCG introduced a health optimisation policy on access to hip replacement surgery. We considered ≥ 18 months of data post-policy introduction as sufficient to give time for policy implementation and possible influence of existing waiting lists. CCGs were excluded where their policy start date was unknown, policies were stopped and restarted or where insufficient post-policy introduction data were available. Details of the policy for each CCG included in the analyses are provided in Additional file 2.

### Control

Each CCG that introduced a policy, acted as its own control, through a comparison of trends in rates of surgery in the time period before policy introduction and the time period after it was introduced. To account for potential external influencing factors, data from CCGs with no policy introduction over the time period of interest were included to control for secular changes in outcomes, using a difference-in-differences controlled interrupted time series study design [20]. This approach provides a test of the differential effects of the intervention time point between the intervention and control groups.

### Effect modification variables

To explore the heterogeneity according to the type of CCG BMI policy, policies were categorised as 1 (mild—patients receive advice only), 2 (moderate—patients are subject to additional waiting time before surgery) or 3 (strict—patients must be below a BMI threshold to be eligible for surgery).

### Statistical analyses

We began by using interrupted time series analysis to examine the impact of policy introduction on trends in the quarterly rates of hip replacement surgery for each CCG that introduced a policy. Segmented linear regression models were used to estimate the trend before policy introduction, and how this trend changed after policy introduction, also allowing for an immediate step change at the date the policy was introduced [20]. The post-intervention counterfactual was estimated as the continuation of the pre-policy introduction period trend. Initial visual assessment of these graphs of quarterly rates during the study period showed no ‘level change’ in the rates of operations evident after policy introduction. Instead, differences in the slope of rate changes post-policy introduction were observed in intervention CCGs. This was considered the ‘effect size’. Random effects meta-analysis was used to pool the change in slope across CCG groups, stratifying according to whether the CCG policy was mild, moderate or strict.

Data on the rates of surgery for all intervention CCGs were then pooled, with the policy introduction date in each CCG being considered time ‘0’ for the sake of alignment. A single-segmented linear regression model was then fitted to obtain an overall national estimate of the impact of health optimisation policy introduction in England. To control for secular effects, non-policy control CCGs were randomly matched to policy CCGs and assigned their policy start date. Both policy and non-policy CCG data were then pooled, and the difference between the rate of hip replacement surgery in intervention and control CCG groups was calculated for each quarter. A controlled interrupted time series analysis was conducted using segmented linear regression of the differences between the groups [20], to compare the differences in trends and estimate an overall national effect of intervention compared to control CCGs. The Newey-West standard error model was used to address the autocorrelation in the data detected with the Durbin-Watson test [30, 31].

Interrupted time series analyses were completed with the same methodology using the secondary outcome measures of the proportion of independently funded operations, the proportion of operations performed in patients with obesity (BMI 30 +) and the mean pre-operative Oxford Hip Score.

Stratifications of the trends in surgery data for the time series analyses were also conducted by policy severity categories.

All statistical analyses were conducted using Stata/MP version 16.1. The analyses were developed and reported according to the RECORD extension [32] to STROBE guidelines for observational studies using routinely collected data (Additional file 1: Table S1).

### Patient and public involvement

The Patient Experience Partnership in Research (PEP-R) group is a regional facilitated group [33], most of whom have had joint replacement, that provides patient and public input into research. Through engagement with PEP-R in preparation for the proposal of this research, the group communicated the opinion that it is ‘vital to provide patients with evidence for the benefits of these policies if they are to be used’. Further engagement with the group during study design and analysis shaped the categorisation of policy severity. The group will also be engaged in planning the dissemination of the study results.

## Results

### Descriptive information and demographics

Of the 181 CCGs in continuous existence from 2013 to 2019, 46 (25.4%) were excluded due to incomplete policy information or complex policy activity timelines (e.g. stops and starts to policy use). One hundred and thirty CCGs were included in the analyses, of which 74 (56.9%) had no policy (control CCGs) and 56 (43.1%) had a policy (intervention CCGs). Of those with policies, 26 (46.4%) had mild (advice only) policies, 14 (25.0%) had moderate (extra waiting time) policies and 16 (28.6%) had strict (mandatory BMI threshold) policies. Policy introduction dates ranged from mid-2013 to mid-2018. A descriptive summary of the range and trend in policy position for CCGs is reported by McLaughlin et al. [8]; there is heterogeneity in the BMI value applied in BMI thresholds (range 25 to 45 kg/m^2^) and in the length of the extra waiting time enforced (range 3 to 12 months). Additional file 2: Table S2 details the CCGs included in the analysis, their policy types and start dates. Additional file 3: Fig. S1 provides the data flowchart for the analysis.

Within these CCGs, a total of 480,364 patients aged 40 + years had a primary hip replacement between January 2009 and December 2019 in England, with osteoarthritis as a primary reason for surgery. The mean age of patients was 68.9 years (SD 10.4), and 290,996 (60.6%) were women. BMI was not recorded for 26.3% of patients. The mean BMI of patients with a BMI record was 28.6 kg/m^2^ (SD 5.23), 415,550 (86.5%) operations were publicly funded and 23,398 (4.9%) patients who received operations were from the 10% of most deprived areas.

The overall rates of surgery increased over time from 41.6 per 100,000 population aged 40 + per quarter year in 2009 to a peak of 72.6 in 2018, before declining to 59.5 in 2019. This pattern was consistent across intervention and control CCG localities. There were approximately 11,000 operations in each quarter in total (mean 10,775, range 7889 to 13,581).

### Baseline differences between the intervention and control CCG groups

Intervention group CCGs had higher mean rates (per 100,000 aged 40 +) of surgery at the start of the time period (2009 quarter 2) than the control group CCGs: 45.5 (SD 16.8) compared to 34.7 (SD 16.9). Table 1 shows the differences between the groups when ‘baseline’ is considered to be 18 months before the policy introduction date. In CCGs that went on to introduce policies, their patient cohorts were similarly obese to CCGs without policies, but their cohorts were more affluent and had more independently funded operations. These differences in characteristics of the CCGs were sustained over time; CCGs choosing to introduce a BMI policy had higher rates of hip replacement and operated on a lower proportion of patients from the most socio-economically deprived areas (quintile 1) at all points in calendar time (Additional file 4: Fig. S2).

**Table 1:** Operation rate and patient characteristics of intervention and control CCGs pre- and post-policy introduction

Operation and patient characteristics	Control CCGs (no policy introduced during the study period)			Intervention CCGs (policy introduced during the study period)		
	Baseline 18 months pre	18 months post	3 years post	Baseline 18 months pre	18 months post	3 years post
	*N* = 74	*N* = 74	*N* = 37	*N* = 56	*N* = 56	*N* = 30
Hip replacement operations rate per 100,000 population aged 40 + years per quarter (mean)	57.6	54.1	55.4	62.2	65.7	62.9
Age (mean)	68.4	68.1	68.3	68.8	68.6	69.1
Gender (% male)	40.3%	42.5%	40.6%	39.0%	39.8%	37.7%
BMI missing (%)	33.9%	37.0%	36.0%	26.4%	25.1%	28.7%
BMI (mean kg/m^2^)	28.6	28.4	28.9	28.3	28.6	28.3
Underweight: BMI below 18 kg/m^2^ (%)	0.3%	0.7%	0.4%	0.3%	0.7%	0.7%
Healthy weight: BMI 18 to 24.9 kg/m^2^ (%)	21.5%	22.7%	20.1%	23.6%	22.2%	24.9%
Overweight: BMI 25 to 29.9 kg/m^2^ (%)	38.9%	40.7%	38.9%	39.9%	38.0%	34.4%
Obese category 1: BMI 30 to 34.9 kg/m^2^ (%)	26.3%	22.9%	25.7%	24.1%	26.4%	27.5%
Obese category 2: BMI 35 to 39.9 kg/m^2^ (%)	9.7%	9.7%	10.3%	9.3%	8.9%	9.7%
Obese category 3: BMI 40 + kg/m^2^ (%)	0.03	3.3%	4.6%	2.9%	3.8%	2.7%
Independently funded surgery (%)	12.2%	11.8%	10.1%	15.5%	15.6%	16.8%
ASA^a^ grade (mean)	2.06	2.05	2.06	2.04	2.04	2.03
1—normal health (%)	12.9%	12.8%	12.5%	13.1%	12.6%	13.6%
2 (%)	68.4%	70.2%	69.9%	70.2%	70.8%	69.9%
3, 4 or 5—poorest health (%)	18.7%	17.0%	17.6%	16.6%	16.6%	16.5%
Index of multiple deprivation (mean score)	16,672	16,492	16,388	19,001	19,215	20,317
Most deprived 20% (quintile 1)	17.3%	17.4%	18.9%	11.7%	10.2%	7.2%
More deprived 20–40%	22.2%	21.8%	21.1%	15.9%	15.8%	15.4%
Mid 20% deprived	19.2%	21.3%	19.0%	21.3%	22.7%	21.3%
Less deprived 20–40%	22.3%	21.4%	23.5%	25.2%	24.3%	24.1%
Least deprived 20% (quintile 5)	18.9%	18.1%	17.5%	25.9%	26.9%	32.0%
Pre-op Oxford Hip Score (mean)	16.9	17.6	17.6	18.1	18.5	18.4
Post-op Oxford Hip Score (mean)	38.4	38.8	38.1	39.6	39.5	39.4
Difference in pre to post-op score (mean)	21.5	21.3	20.6	21.5	21.0	21.0

^a^American Society of Anesthesiologists

### Primary outcome in intervention CCGs: patterns in the rate of surgery following policy introduction

Interrupted time series analysis for individual CCGs in the intervention group (*n* = 56) showed heterogeneity in the effect of policy introduction on the rate of hip replacement operations. Where a change in trend was observed, it was consistent with the time point of policy introduction identified a priori. The immediate change in slope observed after policy introduction for each CCG was independent of differences in the date of policy introduction (e.g. the same effect was observed for a CCG introducing a policy in 2014, as for a CCG introducing the policy in 2018). The effect sizes ranged from a change in post-introduction from the pre-introduction trend in the rate of operations of − 1.85 to + 2.86. Seven of the 16 (43.8%) strict policy CCGs, eight of the 14 (57.1%) moderate policy CCGs and 11 of the 26 (42.3%) mild policy CCGs had a decrease in the rate of operations following policy introduction (effect size estimate < 0). Two CCGs (3.6%), one mild and one strict, had an increase in the rate of operations (effect size estimate 95% C.I lower bound > 0).

In a meta-analysis (random effects), the overall effect size of policy introduction was − 0.00 (95% CI − 0.20 to 0.20) operations per quarter per 100,000 patients aged 40 + years. The effect size was associated with policy severity; in meta-analysis within policy categories, the effect size was − 0.17 (95% CI − 0.57 to 0.23), − 0.07 (95% CI − 0.48 to 0.33) and 0.17 (95% CI − 0.12 to 0.46) operations per quarter per 100,000 patients aged 40 + years in strict, moderate and mild policies, respectively (Additional file 5: Fig. S3).

### Comparison of outcomes in control and intervention CCGs

**Fig. 1:**
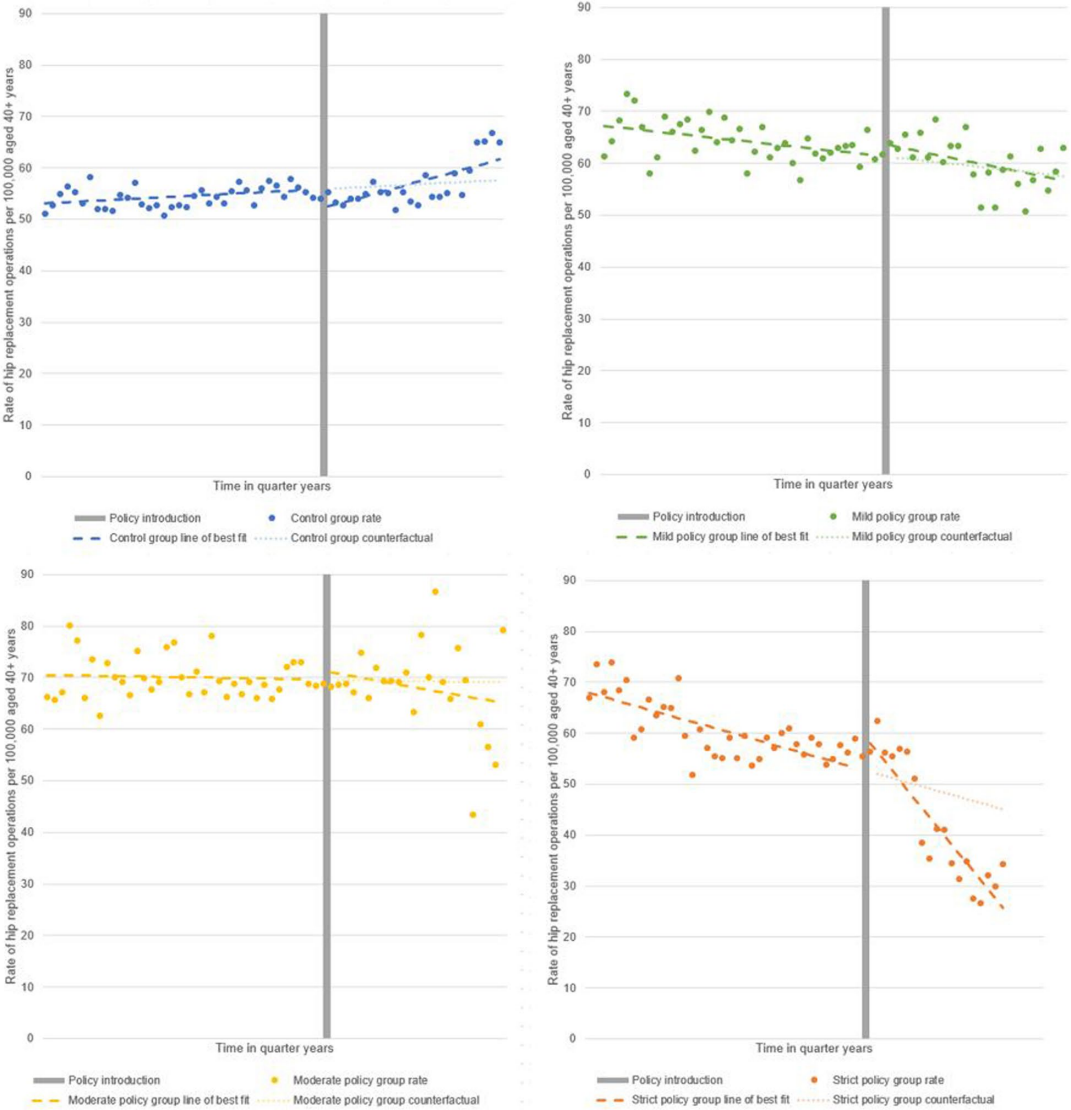
Interrupted time series analyses of hip replacement rates by body mass index policy severity. Rate of hip replacement operations per 100,000 population aged 40 + , per quarter by the level of severity of body mass index policy; none (*n* = 74), mild (*n* = 26), moderate (*n* = 14) and strict (*n* = 16)

The interrupted time series analyses of the rate of hip replacement operations per 100,000 population aged 40 + , per quarter for pooled data by level of severity of body mass index policy are presented in Fig. 1. It illustrates the trend in operation rates pre- and post-policy introduction for the control and intervention CCGs, including by stratification of policy severity.

From the point of policy introduction, control group CCGs had no overall directional change in their trend; the rate of surgery continued to increase over time. There was an association with an increase in the upward trend in the post-policy introduction period (*p* = 0.007).

In contrast, for the intervention CCGs, there was a downward trend in the rate of surgery over time. This accelerated at the point of policy introduction and was then sustained over time resulting in the mean rate of surgery becoming lower for intervention CCGs than for control CCGs. The most pronounced change was observed in the group of CCGs with the strictest BMI policy.

Table 2 presents the interrupted time series segmented linear regression model outputs for the control and policy categories of intervention CCGs. The largest change in trend from the pre- to post-policy introduction period was for the strict policy CCGs: trend change − 1.39 per quarter, 95% confidence interval (CI) − 1.81 to − 0.97, *P* < 0.001. There was no equivalent post-policy introduction change evident in the mild and moderate policy CCG groups. When the strict policy group was compared to the control group in difference-in-differences analysis, the difference in operation rates between the groups widens consistently over time; by − 2.43 (95% CI − 2.86 to − 2.01, *P* < 0.001) operations per 100,000 aged 40 + per quarter in the post-policy introduction period (Table 2).

**Table 2:** Segmented linear regression and difference-in-difference analyses before and after policy introduction

		**Pre-policy** **introduction period**			**Post-policy** **introduction period**					
**Outcome**		Quarterly trend	95% CI		Quarterly trend	95% CI		Change in quarterly trend compared to pre-intervention	95% CI	
**Rate of hip replacement surgery in 100,000 population aged 40 + years**	Control	0.07	− 0.01	0.14	0.40	0.16	0.63	0.32	0.09	0.56
	Mild	− 0.16	− 0.30	− 0.02	− 0.29	− 0.50	− 0.09	− 0.14	− 0.37	0.10
	Moderate	− 0.02	− 0.17	0.13	− 0.26	− 0.75	0.24	− 0.23	− 0.74	0.27
	Strict	− 0.41	− 0.54	− 0.27	− 1.80	− 2.22	− 1.34	− 1.39	− 1.81	− 0.97
	Difference in differences; strict rate minus control rate	− 0.48	− 0.60	− 0.37	− 2.91	− 3.67	− 2.15	− 2.43	− 3.17	− 1.69

### Changes in patient characteristics after policy introduction

Changes in patient characteristics were associated with policy introduction in intervention CCGs compared to control CCGs, indicating a differential impact of policies on different patient groups. Table 1 presents the patient characteristics in the CCGs at baseline, at 18 months post-policy introduction and at 3 years post-policy introduction. Patients in intervention CCGs were more likely to be less deprived, independently (privately) funded and healthy weight at baseline, and these differences were maintained into the post-introduction period. The ‘policy introduction date’ for control CCGs was the date of policy introduction from a randomly paired intervention CCG.

**Fig. 2:**
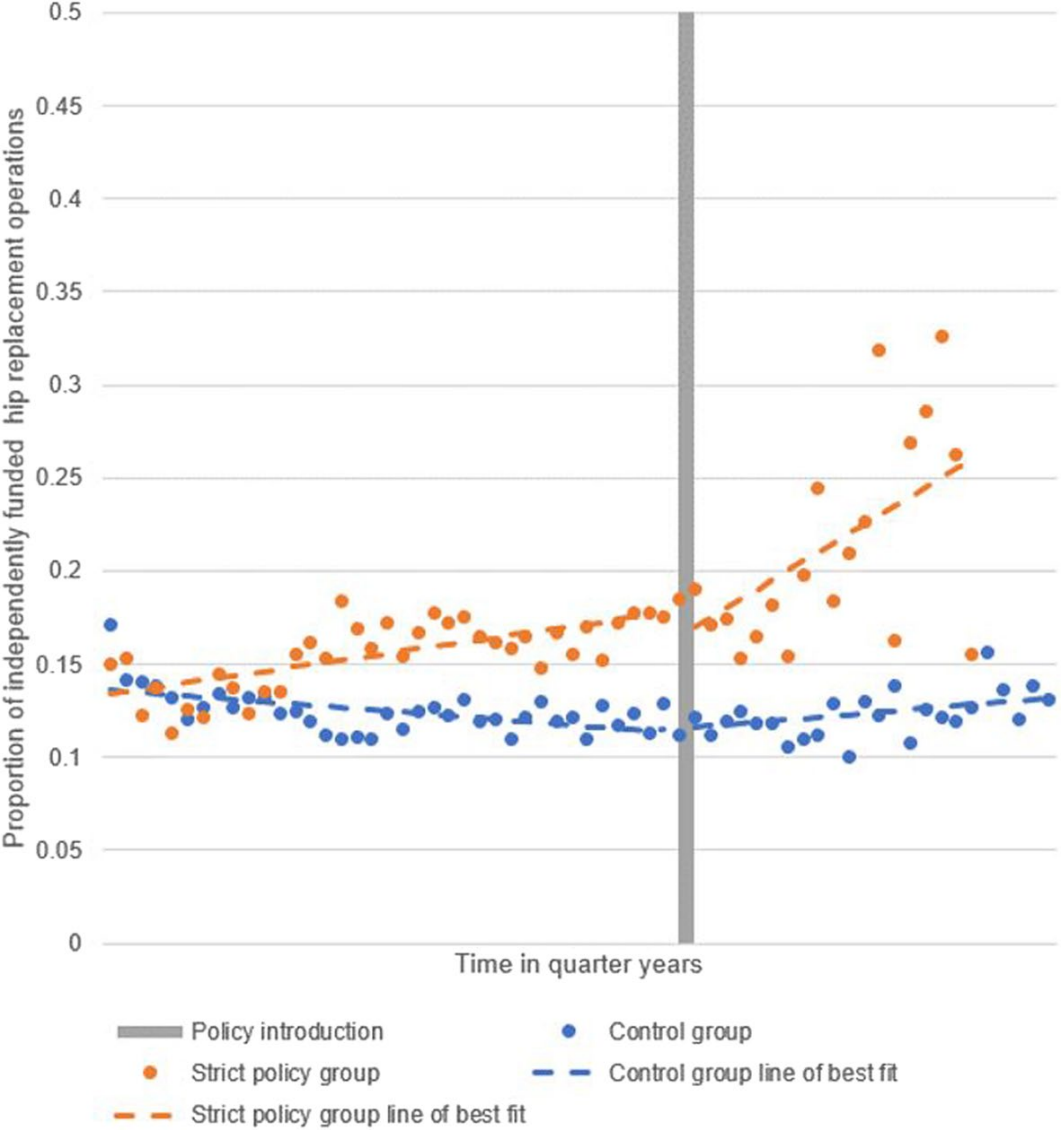
Interrupted time series of proportion of independently funded hip replacement operations. Pooled data for strict policy CCGs (*n* = 16) and control CCGs (*n* = 74)

Figure 2 presents the interrupted time series analysis of the proportion of independently funded operations performed between the control and strict policy groups. While the strict policy group showed an upward trend in the proportion of independently funded surgery even in the pre-policy introduction period, the point of policy introduction was associated with a stronger, sustained upturn in the proportion. For illustration, at 3 years post-policy introduction, the proportion of independently funded surgery in the strict policy group is over double that of the control group (21.0% (SD 7.4%) and 10.1% (SD 9.5%), respectively).

**Fig. 3:**
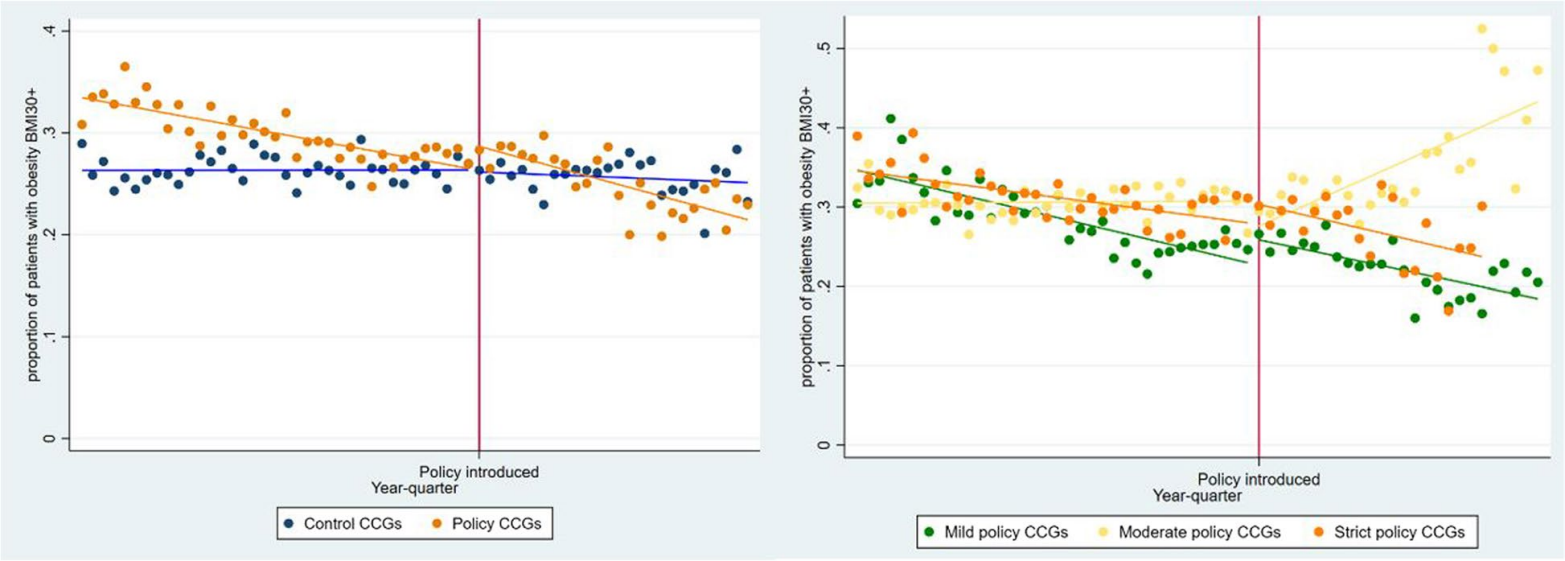
Interrupted time series of proportion of operations where the patient had obesity (BMI 30 + kg/m^2^). Pooled data for **(left)** intervention CCGs (*n* = 56) and control CCGs (*n* = 74) and **(right)** stratified by policy severity

Figure 3 presents the interrupted time series analysis for the proportion of operations performed in patients with obesity (BMI 30 + kg/m^2^). The proportion in the control group remained at approximately 26%, whereas the proportion in the intervention CCGs was higher in the pre-policy period but followed a downward trend into the post-policy introduction period. When the intervention group CCG analyses are stratified by policy severity, the reduction in the intervention group is shown to be driven by reductions in the mild and strict policy types. In contrast, following policy introduction in the moderate (extra waiting time) policy group, there is an association with an increase in trend in this proportion.

**Fig. 4:**
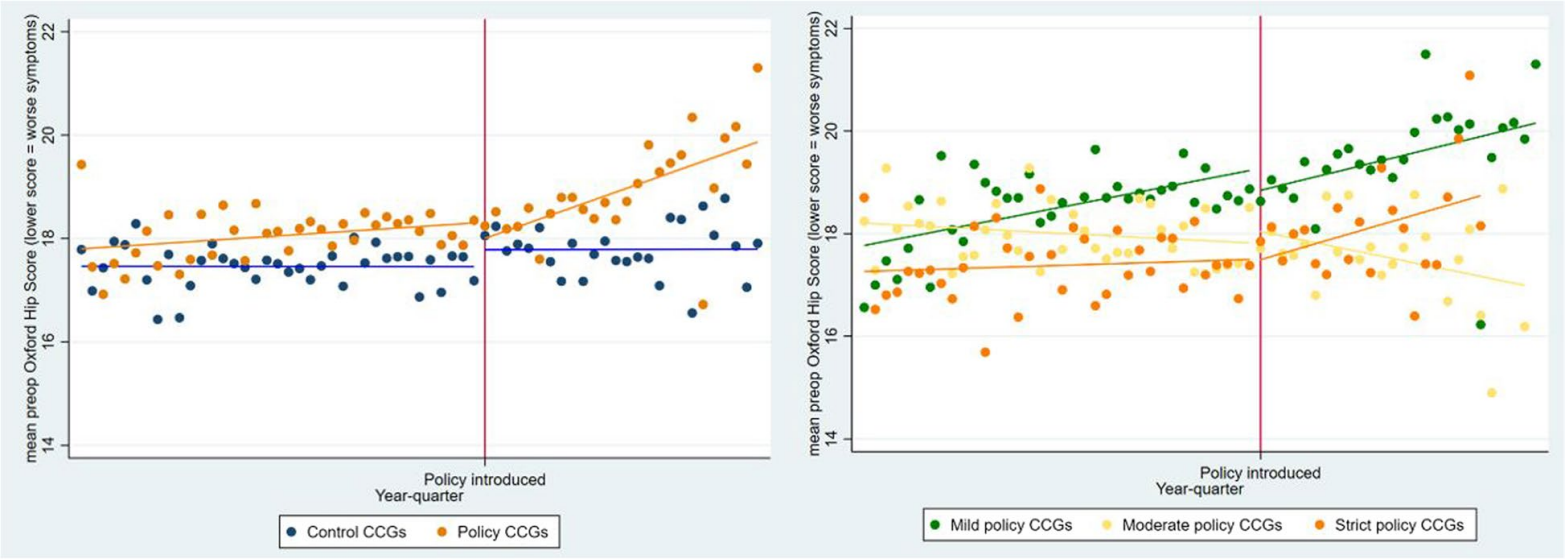
Interrupted time series of mean preop Oxford Hip Score (lower score = worse symptoms). Pooled data for **(left)** all intervention CCGs (*n* = 56) and control CCGs (*n* = 74) and **(right)** stratified by policy severity

Figure 4 presents the interrupted time series analysis for the mean Oxford Hip Score measured pre-operatively in operations performed. The mean score in the control group remained at approximately 17, whereas the mean score in the intervention CCGs was already higher (indicating less severe symptoms) in the pre-policy period and showed an upturn in the trend in the post-policy introduction period. When the intervention group CCG analyses are stratified by policy severity, the increasing trend in the intervention group is shown to be driven by reductions in the mild and strict policy types. In contrast, following policy introduction in the moderate (extra waiting time) policy group, there is a decrease in the trend of the mean score.

## Discussion

The introduction of strict policies requiring patients with obesity to engage with weight loss to access hip replacement surgery was associated with a reduction in the rate of surgery that was sustained over time. Changes in the rate of surgery were less pronounced for mild or moderate BMI policies and opposite to that seen in control CCGs with no policy. This study used observational data to examine the changes in surgery rates and patient characteristics; however, the pooling of data from 130 CCGs, including control CCGs, and the variation in the dates of policy introduction make this a robust natural experiment [23].

Clinical commissioning groups which introduced BMI policies had higher rates of surgery and more affluent populations at baseline compared to those which did not, and it is possible that these factors may have been drivers for policy introduction. Strict policy introduction was associated with an increase in the proportion of independently funded surgery and the proportion of more affluent patients receiving surgery. These findings raise the concern that the use of BMI policies for hip replacement surgery risks widening health inequalities by increasing the link between access to surgery and socioeconomic circumstance, in line with our previous findings regarding knee replacement surgery [19].

The interpretation of a reduction in the rate of surgery may be positive or negative in nature. BMI policies may have reduced the need for surgery for some patients where successful weight loss provided significant relief of their hip symptoms. However, considering that literature reports low rates of success with weight loss efforts and maintenance (an average of 3% weight loss in adults adhering to lifestyle weight loss programmes and weight regain common at one year [34–36]) and a recommendation for at least a 10% reduction in body weight for osteoarthritis patients with obesity to gain meaningful relief in their arthritis outcomes [37], this number is likely to be small. An alternative, less positive explanation for the reduction in the rate of surgery would be that the BMI policies prevent access to surgery by some patients who would have received benefits to their quality of life from hip replacement but were unable to lose sufficient weight. This explanation is supported by literature from the USA reporting that very few patients denied joint replacement due to their obesity manage to lose sufficient weight to qualify for surgery [38].

There is some evidence from this study that BMI policies that impose extra waiting time on patients are counterproductive in certain key measures; patterns in the post-policy introduction period suggest that this type of policy introduction was associated with worsening symptoms (pre-operative Oxford Hip Score) and increasing obesity in the surgical patient population. Existing literature shows evidence that waiting longer for elective surgery gives worse outcomes and loss of quality of life [39]. The proportion of patients with obesity was seen to decrease in the mild and strict policy categories, though it is noted that this was a pre-existing trend.

The rise in surgery rates in the control CCG groups over time is consistent with expectations of a greater need for surgery in an ageing and increasingly obese population in England [5]. The introduction of a moderate or strict policy in one CCG may also result in the referral of affected patients to neighbouring CCGs with less severe policies, raising pressure on their service provision. This may account for some of the rise seen in the control group. The number of patients on existing waiting lists before policy implementation may influence the timing of policy impact, but this association could not be analysed in this study. We are undertaking an associated qualitative study with key professional informants to provide explanatory background on the intended and observed effects of BMI policies for joint replacement [40].

The use of the National Joint Registry is a strength of this study as it captures 96% of all hip replacement procedures including those that are independently funded [41], and for this study, the IMD 2015 was linked to all patients. BMI and patient-reported outcome measure data are less complete in the registry—missing for approximately 25% and 66% of records, respectively. Some surgery eligibility policies included restrictions on patients who smoke. As the NJR does not collect data on smoking status, no analysis was possible on this. Analysis of changes in the rates of surgery gives important insight into the impact of BMI policy introduction, but further research is needed to determine the mechanism of effect and the impact on the quality of life of patients who did not receive a surgical referral.

This study strengthens the evidence for the assertion in the newly updated National Institute for Health and Care Excellence guidelines for Osteoarthritis [42] which state that BMI should not be used to deny patients access to hip replacement surgery, particularly as ‘osteoarthritis is more common in people in lower socio-economic groups. Obesity is also more common in people in lower socio-economic groups and access to surgery on the basis of BMI has been raised by stakeholder groups as an important equality issue’ [43].

NHS commissioning has now moved from CCGs to Integrated Care Boards in England, and it remains to be seen what action they will take where they have inherited strict policies from their former CCGs. Our associated study on knee replacement surgery [19] reflects similar findings and concerns in this patient group, and other elective surgery pathways should be examined for BMI policy use.

## Conclusions

It is our recommendation that BMI policies involving extra waiting time or mandatory BMI thresholds are no longer used to reduce access to hip replacement surgery. Commissioners and policymakers should note the counterproductive effects of policies that deliberately delay access to surgery and the widening of health inequalities, since the ability to pursue independently funded surgery ranges with patients’ affluence.

## Supplementary Information

**Additional file 1:** **Table S1.** The RECORD statement – checklist of items, extended from the STROBE statement, that should be reported in observational studies using routinely collected health data. 

**Additional file 2: Table S2.** Details of clinical commissioning group policies on weight loss and body mass index thresholds for hip replacement surgery for CCGs in existence from Jan 2013 to Dec 2019. Policies started less than 18 months prior to Dec 2019 are not included.

**Additional file 3: Fig. S1.** Flowchart of data included in the analysis.

**Additional file 4: Fig. S2.** Changes incalendar time of rate of hip replacement operations per 100,000 population aged 40+, per quarter andof proportion of patients from the most socio-economically deprived areasfrom pooled data for all intervention CCGsand control CCGs.

**Additional file 5:  Fig. S3.** Forest plot of policy introduction effect size by policy categoryand with overall meta-analysis result for the intervention CCGs.

## Data Availability

Access to data is available from the National Joint Registry for England and Wales, Northern Ireland and the Isle of Man, but restrictions apply to the availability of these data, which were used under licence for the current study, and so are not publicly available. Data access applications can be made to the National Joint Registry Research Committee. Additional file 2: Table S2 contains the data regarding CCG policy introduction dates and levels of severity.

## Abbreviations

ASA: American Society of Anesthesiologists
BMI: Body mass index
CCG: Clinical commissioning group
CI: Confidence interval
IMD: Index of multiple deprivation
LSOA: Lower Layer Super Output Areas
NHS: National Health Service
NJR: National Joint Registry for England, Wales, Northern Ireland and the Isle of Man
OECD: Organization for Economic Cooperation and Development
PEP-R: Patient Experience Partnership in Research
PROMS: Patient-reported outcome measures
SD: Standard deviation
