# Supplementary material for: What effect have commissioners’ policies for body mass index had on hip replacement surgery?: an interrupted time series analysis from the National Joint Registry for England
Source: BMC Med. 2023 Jun 13;21:202. doi: 10.1186/s12916-023-02899-3 (PMC10260274; doi:10.1186/s12916-023-02899-3)
Supplement: Supplementary file 2 — Additional file 2: Table S2. Details of clinical commissioning group policies on weight loss and body mass index thresholds for hip replacement surgery for CCGs in existence from Jan 2013 to Dec 2019. Policies started less than 18 months prior to Dec 2019 are not included. [file 12916_2023_2899_MOESM2_ESM.pdf]

Supplementary Table 2: Details of clinical commissioning group policies on weight loss and body mass index thresholds for hip replacement surgery for CCGs in existence from Jan 2013 to Dec 2019. Policies started less than 18 months prior to Dec 2019 are not included.

CCG	CCG Name	Policy category 0-3**	Policy start date m/d/y
E38000004	NHS BARKING AND DAGENHAM CCG	0	
E38000005	NHS BARNET CCG	0	
E38000006	NHS BARNSLEY CCG	2	01/01/2018
E38000007	NHS BASILDON AND BRENTWOOD CCG	0	
E38000010	NHS BEDFORDSHIRE CCG	1	02/01/2016
E38000011	NHS BEXLEY CCG	0	
E38000014	NHS BLACKBURN WITH DARWEN CCG	0	
E38000015	NHS BLACKPOOL CCG	0	
E38000016	NHS BOLTON CCG	0	
E38000020	NHS BRENT CCG	0	
E38000021	NHS BRIGHTON AND HOVE CCG	2	04/01/2018
E38000023	NHS BROMLEY CCG	0	
E38000024	NHS BURY CCG	0	
E38000025	NHS CALDERDALE CCG	0	
E38000026	NHS CAMBRIDGESHIRE AND PETERBOROUGH CCG	0	
E38000027	NHS CAMDEN CCG	0	
E38000029	NHS CANTERBURY AND COASTAL CCG	1	12/01/2017
E38000030	NHS CASTLE POINT AND ROCHFORD CCG	0	
E38000031	NHS CENTRAL LONDON (WESTMINSTER) CCG	0	
E38000034	NHS CHORLEY AND SOUTH RIBBLE CCG	2	11/01/2017
E38000035	NHS CITY AND HACKNEY CCG	0	
E38000037	NHS CORBY CCG	0	
E38000038	NHS COVENTRY AND RUGBY CCG	3	02/01/2016
E38000040	NHS CROYDON CCG	1	04/01/2013
E38000043	NHS DARTFORD GRAVESHAM AND SWANLEY CCG	1	12/01/2017
E38000044	NHS DONCASTER CCG	2	01/01/2017
E38000045	NHS DORSET CCG	1	06/01/2017
E38000048	NHS EALING CCG	0	
E38000050	NHS EAST LANCASHIRE CCG	0	
E38000051	NHS EAST LEICESTERSHIRE AND RUTLAND CCG	3	11/01/2017
E38000053	NHS EAST STAFFORDSHIRE CCG	1	11/01/2017
E38000054	NHS EAST SURREY CCG	1	01/01/2016
E38000057	NHS ENFIELD CCG	0	
E38000059	NHS FAREHAM AND GOSPORT CCG	0	
E38000062	NHS GLOUCESTERSHIRE CCG	2	10/01/2015
E38000064	NHS GREATER HUDDERSFIELD CCG	0	
E38000066	NHS GREENWICH CCG	0	
E38000068	NHS HALTON CCG	3	06/01/2017
E38000069	NHS HAMBLETON RICHMONDSHIRE AND WHITBY CCG	0	
E38000070	NHS HAMMERSMITH AND FULHAM CCG	3	11/01/2014

E38000072	NHS HARINGEY CCG	0	
E38000073	NHS HARROGATE AND RURAL DISTRICT CCG	2	10/01/2016
E38000074	NHS HARROW CCG	3	11/01/2014
E38000077	NHS HAVERING CCG	0	
E38000078	NHS HEREFORDSHIRE CCG	0	
E38000080	NHS HEYWOOD, MIDDLETON AND ROCHDALE CCG	0	
E38000081	NHS HIGH WEALD LEWES HAVENS CCG	0	
E38000082	NHS HILLINGDON CCG	3	11/01/2014
E38000084	NHS HOUNSLOW CCG	3	11/01/2014
E38000085	NHS HULL CCG	2	08/01/2016
E38000087	NHS ISLE OF WIGHT CCG	0	
E38000088	NHS ISLINGTON CCG	0	
E38000089	NHS KERNOW CCG	0	
E38000090	NHS KINGSTON CCG	1	04/01/2013
E38000091	NHS KNOWSLEY CCG	0	
E38000092	NHS LAMBETH CCG	0	
E38000097	NHS LEICESTER CITY CCG	3	11/01/2017
E38000098	NHS LEWISHAM CCG	0	
E38000099	NHS LINCOLNSHIRE EAST CCG	1	06/01/2013
E38000100	NHS LINCOLNSHIRE WEST CCG	1	06/01/2013
E38000101	NHS LIVERPOOL CCG	0	
E38000102	NHS LUTON CCG	1	03/01/2017
E38000104	NHS MEDWAY CCG	1	12/01/2017
E38000105	NHS MERTON CCG	1	04/01/2013
E38000106	NHS MID ESSEX CCG	0	
E38000108	NHS NENE CCG	0	
E38000113	NHS NEWHAM CCG	0	
E38000117	NHS NORTH EAST ESSEX CCG	2	09/01/2016
E38000118	NHS NORTH EAST HAMPSHIRE AND FARNHAM CCG	0	
E38000119	NHS NORTH EAST LINCOLNSHIRE CCG	0	
E38000120	NHS NORTH HAMPSHIRE CCG	0	
E38000122	NHS NORTH LINCOLNSHIRE CCG	0	
E38000124	NHS NORTH NORFOLK CCG	3	09/01/2017
E38000127	NHS NORTH TYNESIDE CCG	0	
E38000128	NHS NORTH WEST SURREY CCG	1	01/01/2016
E38000130	NHS NORTHUMBERLAND CCG	0	
E38000135	NHS OLDHAM CCG	0	
E38000136	NHS OXFORDSHIRE CCG	1	09/01/2016
E38000137	NHS PORTSMOUTH CCG	0	
E38000138	NHS REDBRIDGE CCG	0	
E38000140	NHS RICHMOND CCG	1	04/01/2013
E38000143	NHS SALFORD CCG	0	
E38000146	NHS SHEFFIELD CCG	2	07/01/2016
E38000150	NHS SOMERSET CCG	0	
E38000151	NHS SOUTH CHESHIRE CCG	0	
E38000154	NHS SOUTH EASTERN HAMPSHIRE CCG	0	
E38000156	NHS SOUTH KENT COAST CCG	1	12/01/2017

E38000157	NHS SOUTH LINCOLNSHIRE CCG	1	06/01/2013
E38000161	NHS SOUTH SEFTON CCG	0	
E38000163	NHS SOUTH TYNESIDE CCG	0	
E38000164	NHS SOUTH WARWICKSHIRE CCG	3	12/01/2017
E38000165	NHS SOUTH WEST LINCOLNSHIRE CCG	1	06/01/2013
E38000167	NHS SOUTHAMPTON CCG	0	
E38000168	NHS SOUTHEND CCG	0	
E38000170	NHS SOUTHPORT AND FORMBY CCG	0	
E38000171	NHS SOUTHWARK CCG	0	
E38000174	NHS STOCKPORT CCG	0	
E38000176	NHS SUNDERLAND CCG	0	
E38000177	NHS SURREY DOWNS CCG	1	01/01/2016
E38000178	NHS SURREY HEATH CCG	2	01/01/2016
E38000179	NHS SUTTON CCG	1	04/01/2013
E38000180	NHS SWALE CCG	1	12/01/2017
E38000181	NHS SWINDON CCG	2	06/01/2013
E38000182	NHS TAMESIDE AND GLOSSOP CCG	0	
E38000184	NHS THANET CCG	1	12/01/2017
E38000185	NHS THURROCK CCG	0	
E38000186	NHS TOWER HAMLETS CCG	0	
E38000187	NHS TRAFFORD CCG	0	
E38000189	NHS VALE ROYAL CCG	0	
E38000192	NHS WALTHAM FOREST CCG	0	
E38000193	NHS WANDSWORTH CCG	1	04/01/2013
E38000194	NHS WARRINGTON CCG	3	06/01/2017
E38000195	NHS WARWICKSHIRE NORTH CCG	3	04/01/2017
E38000196	NHS WEST CHESHIRE CCG	0	
E38000197	NHS WEST ESSEX CCG	0	
E38000198	NHS WEST HAMPSHIRE CCG	0	
E38000199	NHS WEST KENT CCG	1	12/01/2017
E38000200	NHS WEST LANCASHIRE CCG	0	
E38000201	NHS WEST LEICESTERSHIRE CCG	3	11/01/2017
E38000202	NHS WEST LONDON CCG	3	11/01/2014
E38000203	NHS WEST NORFOLK CCG	2	04/01/2015
E38000205	NHS WIGAN BOROUGH CCG	0	
E38000206	NHS WILTSHIRE CCG	2	03/01/2018
E38000214	NHS GUILDFORD AND WAVERLEY CCG	1	01/01/2016
E38000215	NHS NORTH CUMBRIA CCG	0	
E38000218	NHS NORWICH CCG	3	09/01/2017
E38000219	NHS SOUTH NORFOLK CCG	3	09/01/2017
E38000226	NHS FYLDE AND WYRE CCG	0	
E38000227	NHS GREATER PRESTON CCG	2	11/01/2017
E38000228	NHS MORECAMBE BAY CCG	0	

**Policy categorisation 0-3:

0 - never had a policy

1 - advice only

2 - extra wait

3 - BMI threshold
