# Supplementary figures and images for: What effect have commissioners’ policies for body mass index had on hip replacement surgery?: an interrupted time series analysis from the National Joint Registry for England
Source: BMC Med. 2023 Jun 13;21:202. doi: 10.1186/s12916-023-02899-3 (PMC10260274; doi:10.1186/s12916-023-02899-3)

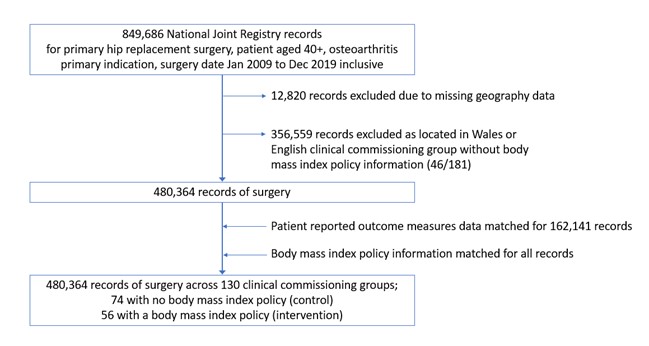

Supplement: Supplementary file 3 — Additional file 3: Fig. S1. Flowchart of data included in the analysis. [file 12916_2023_2899_MOESM3_ESM.jpg]

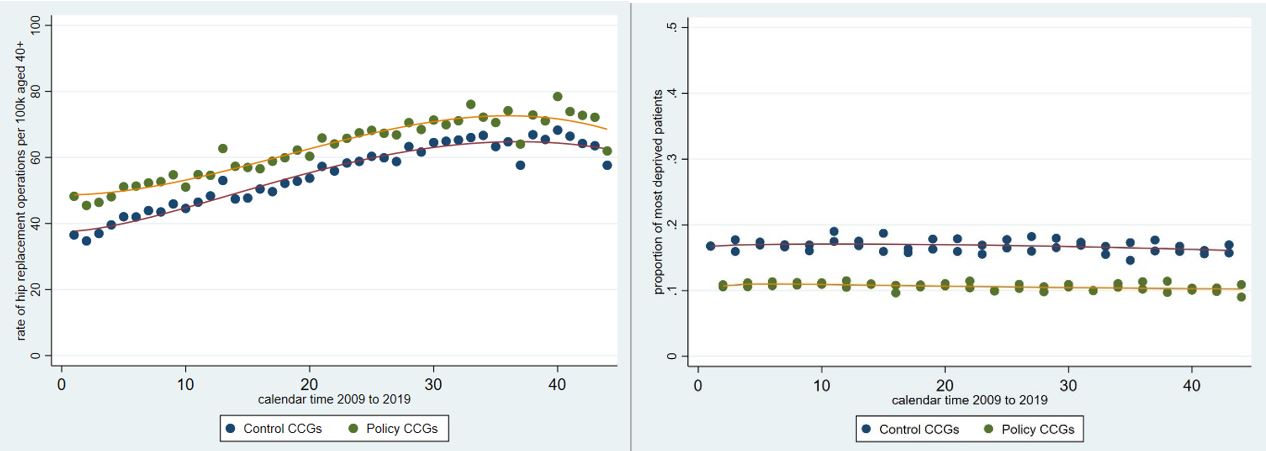

Supplement: Supplementary file 4 — Additional file 4: Fig. S2. Changes incalendar time of rate of hip replacement operations per 100,000 population aged 40+, per quarter andof proportion of patients from the most socio-economically deprived areasfrom pooled data for all intervention CCGsand control CCGs. [file 12916_2023_2899_MOESM4_ESM.jpg]

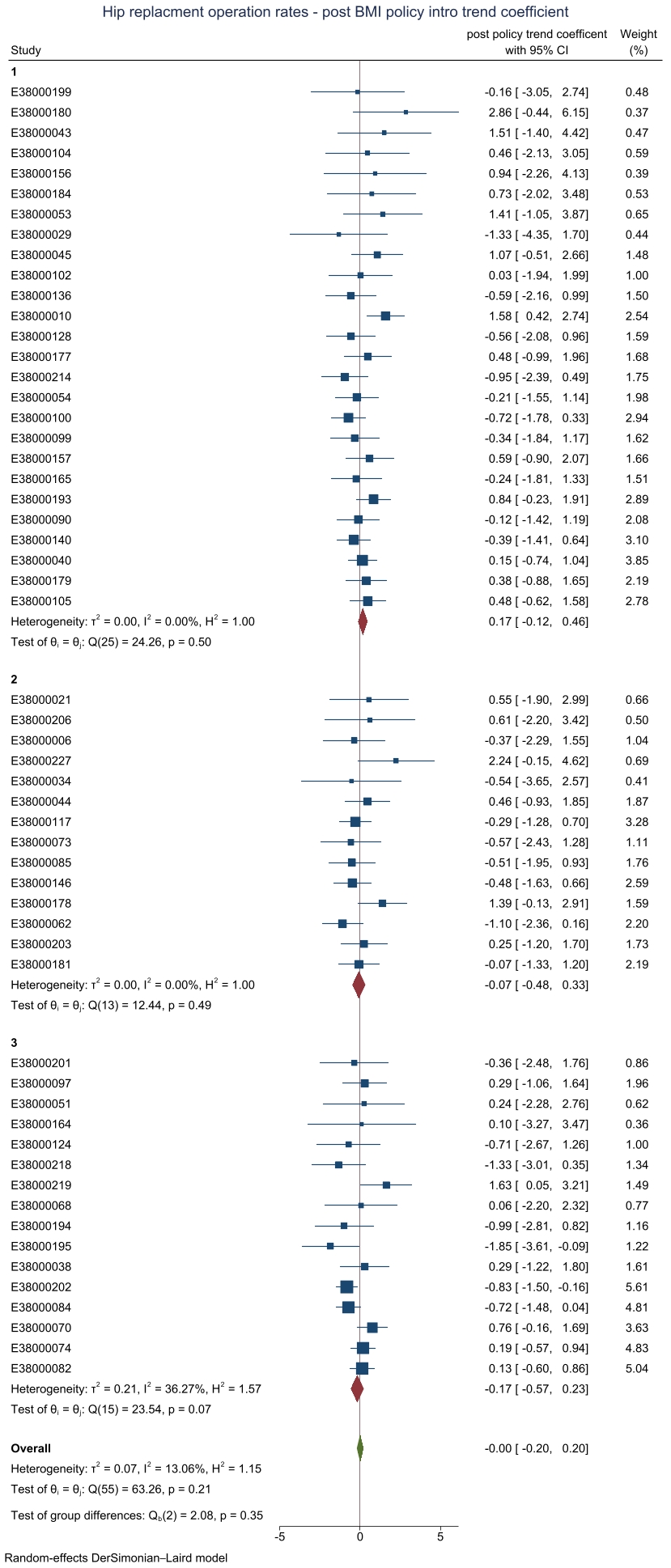

Supplement: Supplementary file 5 — Additional file 5: Fig. S3. Forest plot of policy introduction effect size by policy categoryand with overall meta-analysis result for the intervention CCGs. [file 12916_2023_2899_MOESM5_ESM.jpg]
